# 

**Published:** 1890-03

**Authors:** 


### LITERARY.

We have received since our last issue the following publications for which we desire to make due acknowledgment.

NINETEENTH ANNUAL REPORT of the State Homeopathic Asylum for the Insane, Middletown, N. Y., Selden H. Talcott, M.D., Medical Superintendent. This report shows progress in the right direction. Among its added structures are a new hospital building, a chapel and what is equally important, a Hall for entertainments, all admirably planned and executed.

TRANSACTIONS OF THE MEDICAL ASSOCIATION OF THE STATE OF MISSOURI, year 1889— a compilation in every way worthy of our brethren of the mighty West.

MILLS' ANNUAL CATALOGUE of Garden, Field and Flowers, Thorn Hill, New York. Very complete—profusely illustrated—with a suggested "CURE FOR HARD TIMES" worthy of the especial attention of Farmers and Horticulturists.

OUR LITTLE MEN AND WOMEN AND BABY-LAND. These Juvenile monthlys are without a rival in their line. The March numbers excel all former ones. Don't let your little ones get their eyes on them, if you can't afford to become a subscriber. They will give you no peace. D. Lathrop Co., Boston.

THE ART OF ADVERTISING. Alden & Faxon, Newspaper Advertising Agents, Cincinnati, Ohio, have just issued a very neat List of the leading newspapers of the United States. This firm make a specialty of writing advertisements, and of devising methods by which money can be made out of newspaper advertising. They claim to have been eminently successful in this direction. This Newspaper List will be sent free on application.

